# Deciphering the Combinatorial Roles of Geometric, Mechanical, and Adhesion Cues in Regulation of Cell Spreading

**DOI:** 10.1371/journal.pone.0081113

**Published:** 2013-11-25

**Authors:** Greg M. Harris, Tarek Shazly, Ehsan Jabbarzadeh

**Affiliations:** 1 Department of Chemical Engineering, University of South Carolina, Columbia, South Carolina, United States of America; 2 Department of Mechanical Engineering, University of South Carolina, Columbia, South Carolina, United States of America; 3 Department of Biomedical Engineering, University of South Carolina, Columbia, South Carolina, United States of America; 4 Department of Orthopaedic Surgery, University of South Carolina, Columbia, South Carolina, United States of America; University of California, San Diego, United States of America

## Abstract

Significant effort has gone towards parsing out the effects of surrounding microenvironment on macroscopic behavior of stem cells. Many of the microenvironmental cues, however, are intertwined, and thus, further studies are warranted to identify the intricate interplay among the conflicting downstream signaling pathways that ultimately guide a cell response. In this contribution, by patterning adhesive PEG (polyethylene glycol) hydrogels using Dip Pen Nanolithography (DPN), we demonstrate that substrate elasticity, subcellular elasticity, ligand density, and topography ultimately define mesenchymal stem cells (MSCs) spreading and shape. Physical characteristics are parsed individually with 7 kilopascal (kPa) hydrogel islands leading to smaller, spindle shaped cells and 105 kPa hydrogel islands leading to larger, polygonal cell shapes. In a parallel effort, a finite element model was constructed to characterize and confirm experimental findings and aid as a predictive tool in modeling cell microenvironments. Signaling pathway inhibition studies suggested that RhoA is a key regulator of cell response to the cooperative effect of the tunable substrate variables. These results are significant for the engineering of cell-extra cellular matrix interfaces and ultimately decoupling matrix bound cues presented to cells in a tissue microenvironment for regenerative medicine.

## Introduction

Human mesenchymal stem cells (hMSCs) are uniquely positioned as a highly promising cell source for tissue engineering and cell transplant strategies due to their unique capability of self-renewal and capability to differentiate into many diverse cell types [1], [2], [3], [4], [5], [6], [7]. However, their use as a therapy thus far is hampered due to the limited understanding of mechanisms by which cells integrate environmental stimuli. In the regeneration process, the temporary extracellular matrix (ECM) provides multiple signals to the migrating cells to guide the process of new matrix formation. Major advances have been made in the identification of these biochemical and biophysical regulators of stem cell fate [8], [9], [10], [11], [12], [13], [14], [15], [16], [17], [18]. It has been proposed that many of these signals are intertwined, yet definitive studies have been unable to identify the correlation between biological signaling pathways and how cells receive these signals to develop and repair tissue.

Tissue is fundamentally diverse across ECM environments and plays a major role in cell signaling [19], [20], [21], [22]. The ECM is composed of large amounts of biochemical components including proteins, glycoproteins, proteoglycans, and polysaccharides with vastly different physical and biochemical properties [23], [24]. Cells are able to sense these variances through transmembrane proteins called integrin receptors that help govern cell-ECM signaling and link the cell to the proteins in the ECM [25], [26], [27], [28], [29]. This cell-ECM interaction is crucial to sensing forces through tissue and the surroundings. As early as the 19^th^ century scientists understood physical forces were important to tissue development and were able to show that cultured chick rudiments under static compression following displacement of the periosteum and perichondrium resulted in cartilaginous tissue formation while tensile stresses led to bone formation [30]. More recent studies have uncovered that ECM topography can control cellular organization with the size and geometry of available surface area being able to alter cell shape, traction forces, and cell spreading [12], [31], [32], [33], [34], [35], [36], [37], [38]. Single cell studies further show that smaller ECM islands promote rounded cells while cells in larger islands with no restriction flatten and spread similar to 2D cultures [31], [32]. A key study involving adult stem cells showed micropatterned 10,000 µm^2^ and 1,024 µm^2^ protein areas directed osteogenic differentiation and adipogenic differentiation respectively simply by controlling cell shape and size. Thus, cell shape and size are crucial components in determining stem cell lineage with generally accepted instances of rounded adipocytes [39], [40] and polygonal osteoblasts [41], [42]. Cell shape is highly influenced by ECM elasticity which has the ability to also impact cell spreading, traction forces, cell motility, and differentiation [17], [43], [44], [45], [46], [47], [48], [49], [50]. Researchers have been able to use polyacrylamide gels to mimic tissue elasticity from 1 kPa to 40 kPa and promote differentiation of stem cells into neurogenic, myogenic, and osteogenic lineages through solely altering elasticity [17]. Additionally, matrix elasticity for previously differentiated cells has been shown to alter the cytoskeletal organization as well as the focal adhesion structure [21], [51], [52], [53], [54]. Furthermore, three-dimensional experiments have shown cells capable of migrating and remodeling the ECM in terms of matrix stiffness and topography [55], [56], [57] making it vital to understand the significance of physical signaling and cell-ECM interactions.

A significant step towards further decoupling these signals can be achieved through the development of platforms with tunable physical and topographical properties that allow for further exploration of the co-operative involvement directing cell behavior. While both topography and matrix elasticity have been shown to affect cell morphology independently, there lacks sufficient data correlating these signals. Micropost arrays with varying stiffness and topography pioneered by the Chen laboratory have begun to incorporate the concepts of matrix elasticity with patterning proteins and cell alignment [58], [59], [60], [61], [62], [63]. This research has laid the groundwork to characterize the interplay between physical signals but lacks the ability to change the elastic modulus of the posts, as opposed to stiffness, as well as the elastic modulus of the background ECM. In this preliminary study on deciphering multiple physical cues, we demonstrate a novel method of micropatterning hydrogels to create a tunable matrix with variable elasticity, topography and ligand density as seen in Table 1 and demonstrate how these characteristics affect cell adhesion. A finite element model was also employed to confirm experimental results and utilized as a predictive tool in cell behavior. DPN was employed to micro-pattern islands of poly(ethylene) glycol (PEG) hydrogels onto a polydimethyl siloxane (PDMS) coated surface. DPN is a versatile technique that utilizes a functionalized atomic force microscope tip to transfer molecules of interest to a substrate via a surface meniscus formed between the substrate and tip [64], [65], [66], [67], [68], [69]. Hydrogel islands were patterned onto the PDMS substrates [64] to provide a tunable elasticity and pitch. In this study, we report experimental and modeling results on how the interplay between ECM properties controls cell-adhesion characteristics that define hMSC spreading.

**Table 1:** Table showing the micropatterning characteristics including, background ECM elasticity, island elasticity, island topography, and ligand density values.

Substrate Design	Cell Properties Assessed
Background ECM Elasticity	Cell Area
(12 kPa and 2.5 Mpa)	Focal Adhesion Distribution (Vinculin)
Island Elasticity	Cytoskeletal Organization (F-Actin)
(7 kPa and 105 kPa)	RhoA Signaling Pathway
Island Spacing	
(3 µm, 7 µm, & 12 µm)	
Ligand Density	
(20, 50, & 100 µg ml^−1^)	

Also shown are the characteristics analyzed to determine cell behavior and spreading including cell size, focal adhesion distribution, cytoskeleton arrangement, and RhoA signaling pathway knockdowns.

## Materials and Methods

### Substrate Preparation

Glass coverslips (22×22 mm, Fisher Scientific) were washed with ethanol, dried with nitrogen, and treated for 30 minutes with ozone cleaner (BioforceNano, Ames, IA). PDMS was then spincoated onto cover slips at 500 rotations per minute (RPM) for 10 seconds followed by 2000 RPM for 60 seconds. Cover slips were then sputter coated (Denton Desk II, Moorestown, NJ) with a 5 nm titanium adhesion layer onto PDMS followed by approximately 40 nm of gold.

### Micropatterning of PEG

Islands of PEG hydrogels were patterned using a DPN NSCRIPTOR system with M type pen (Nanoink, Skokie, IL). Pens were ozone treated for 30 minutes prior to inking. PEG precursor was mixed using 700 molecular weight (MW) PEG diacrylate (PEG-DA) (Aldrich, Milwaukee, WI) mixed with 2000 MW 4-arm PEG thiol (PEG-SH)(CreativePEGWorks, Raleigh, NC) in deionized water with 0.5% (v/v) 2-hydroxy-2-methylpropiophenone (Aldrich, Milwaukee, WI). Cover slips were patterned with PEG islands and placed under approximately 4 mW/cm^2^ UV light (UVP, Upland, CA) for 2 minutes to gel. The cover slip was then incubated in 50 mM triethylene glycol mono-mercaptoundecyl ether (Aldrich, Allentown, PA) for 20 minutes to render remaining surface non-adhesive, rinsed with 70% ethanol, and subsequently washed with sterile distilled water three times. Fibronectin (FN) from human plasma (Sigma, St Louis, MO) was incubated at 4°C for 2 hours in heterogenous maleimide/N- hydroxysuccinimide bi-functional linker (ThermoFisher, Rockford, IL) [70] and separated from unreacted linker using a Zeba Spin desalting column (Thermo Fisher, Rockford, IL). Cover slips were then incubated in functionalized FN overnight to allow covalent attachment.

### Hydrogel Characterization

Cylindrical PDMS disks 5 mm in diameter and 5 mm height were fabricated in a 10∶1 and 50∶1 ratio of base to curing agent and let cure for 48 hours at room temperature for differing substrate modulus and sputter coated with titanium and gold layers prior to analysis.[71], [72] PEG hydrogel samples were created 5 mm in diameter and 3 mm height at desired ratio and let soak in deionized water for 48 hours at 37°C. Samples were tested in unconfined compression [73], [74], [75], [76], in short, the Young's modulus of each sample was determined using an ElectroForce 3200 (Bose, Eden Prairie, MN) in unconfined compression at 0.05 mm sec^−1^ between parallel nonporous plates while compressive force and displacement were recorded.

### Cell Culture

Human bone marrow-derived mesenchymal stem cells were obtained from Lonza (Walkersville, NC). hMSCs were cultured in basal growth medium (Lonza, Walkersville, NC) in Nunc cell culture treated 75 cm^2^ flasks (Fisher Scientific). Growth medium contained 440 mL of hMSC basal medium, 50 mL of mesenchymal cell growth supplement, 10 mL of 200 mM L-glutamine, and 0.5 mL of a penicillin/streptomycin mixture. Cells were passaged after reaching 90% confluence and collected with 0.05% trypsin/EDTA solution. All cells were plated onto cover slips under passage 6 at 5,000 cells per cm^2^. Cells were allowed 4 hours for adhesion onto substrates. For ROCK inhibited cells, 10 µM Y-27632 (Calbiochem, Rockaway, NJ) was applied daily for 1 week prior to seeding.

### Immunofluorescent Staining

After incubation for 4 hours in culture medium, cells were fixed with 4% paraformaldehyde, permealized with 0.2% Triton X-100, and blocked with 1% BSA solution. F-Actin, focal adhesions, and nuclei of cells were stained with a rhodamine-phalloidin conjugate (Invitrogen, Grand Island, NY), vinculin (Sigma, St. Louis, MO), and Fluoroshield with Dapi (Sigma, St. Louis, MO) respectively. Fluorescent photographs of hMSCs were captured by a Nikkon Eclipse 80 i microscope with CoolSnap HQ camera. Non-fluorescent cells were analyzed using phase contrast microscopy utilizing NIS-Elements-AR 3.2 64 bit software (NIS-Elements, Melville, NY).

### Simulation Model Analysis

A finite-element model was constructed to quantify the peak deflection of micropatterned substrates in response to cell-derived forces. The model geometry consists of two subdomains, namely a 50 micron thick PDMS substrate and a hemispherical PEG island with a radius of 5 microns. Both PDMS and PEG were modeled as linear elastic, isotropic, incompressible, and homogeneous materials. Model boundary conditions consisted of a 20 nN lateral body force applied to the PEG island, a fixed constraint on the bottom surface of the PDMS substrate, a rigid contact between the PEG and PDMS, and free deformation for all other surfaces. The Poisson's ratio (υ) and density (ρ) of both materials were assigned fixed values, while the elastic moduli (E) were varied in isolation to delineate the effect of substrate and island stiffness on the mechanical behavior. A commercial finite-element software package with a built-in parametric solver (COMSOL) was used to generate stationary solutions to the defined solid mechanics problem. The peak PEG island deflection was extracted from each simulation result and used as a metric of the micropatterned substrate mechanical response to a cell-derived force. A total of 60,177 tertrahedral mesh elements were used to discretize the model geometry and generate mesh-independent solutions, with mesh-independence defined as the level at which further refinement induced a less than 1% change in the predicted peak deflection.

### Statistics

Statistical significance was calculated using one way ANOVA in Excel (Microsoft, Seattle, WA). Linear regression analysis and interaction plot were created using Minitab version 16 software (Minitab Inc., State College, PA). Errors are standard error of the mean.

## Results and Discussion

### Controlling Cell Position and Spreading on Micropatterned ECMs

**Figure 1:**
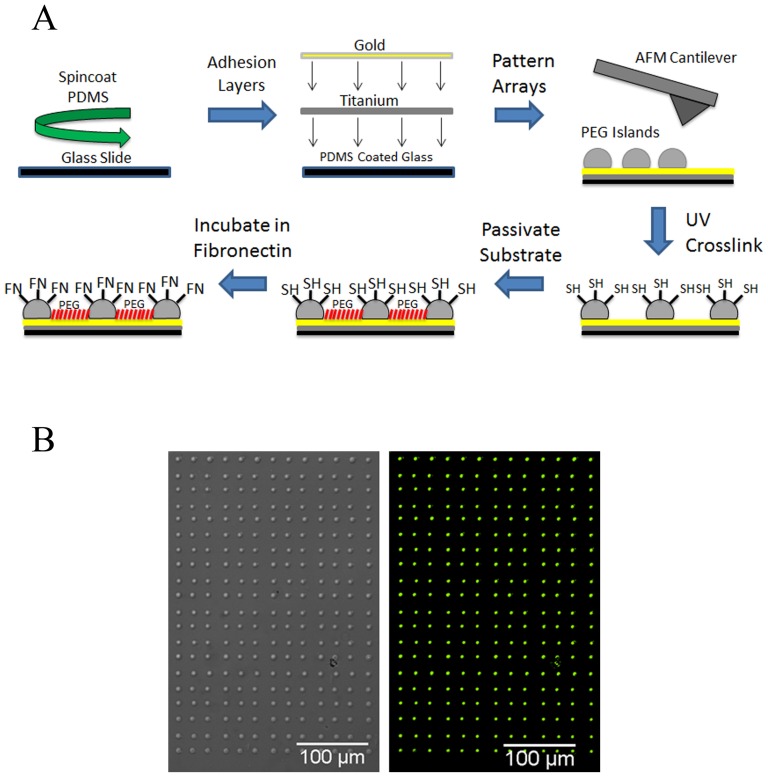
DPN enables micropatterning of sub-cellular hydrogel substrates. (A) PDMS was spincoated onto glass slides to form a background of varying elasticity while utilizing DPN to deposit micropatterned hydrogel islands also with varying elasticity. These sub-cellular islands were functionalized with fibronectin at differing ligand densities, which facilitated cell attachment to substrate. (B) Micropatterned PEG hydrogel islands spaced at 12 µm distance between islands showing 100 µg ml^−1^ BSA-FITC conjugated protein covalently bonded.

In this study we fabricated hydrogel islands using a novel process utilizing DPN to deposit micrometer sized PEG islands onto PDMS coated coverslips as shown in Figure 1A. PEG was chosen due to the non-toxic properties and the ability of this polymer to resist protein adsorption [77], [78]. DPN is a highly versatile technique able to be used in creating islands at differing spacing using a functionalized atomic force microscope tip to directly transfer molecules of interest to a substrate. PEG-DA and PEG-SH mixture was chosen as hydrogel islands and by varying the concentration of PEG precursor, it was possible to more closely mimic the elasticity of tissue at the subcellular level. PEG islands were patterned onto the gold coated PDMS background backfilled with PEG-SH to render the background non-adhesive to protein adsorption and confine cell adhesion to islands. The PDMS background was able to be altered to achieve differing elasticities of the non-adhesive ECM. Preliminary experiments were performed to confirm the ability of proteins to conjugate exclusively to the hydrogel islands, BSA was used as a demonstration protein as shown in Figure 1B. Hydrogel islands were sized at 9.31±0.058 µm in diameter and spaced at 3.15±0.22 µm, 7.09±0.23 µm, and 12.07±0.23 µm pitch to allow cells to spread across multiple islands (50 islands analyzed each case). Hydrogel island elasticities were measured at 7.05±0.72 kPa and 105.07±1.07 kPa respectively. Ligand density was determined by incubating samples in 20 µg ml^−1^, 50 µg ml^−1^, and 100 µg ml^−1^ fibronectin concentrations overnight. PDMS was spincoated onto glass coverslips at a 50∶1 base:curing ratio and a 10∶1 ratio for a differing elasticity of 12±1.0 kPa and 2.5±0.20 MPa respectively [71]. By utilizing a novel micropatterning method we were able to create a tunable array of subcellular hydrogels capable of parsing microenvironmental cues presented to a cell. Attaining this allowed us to successfully integrate geometric, mechanical, and biochemical control in understanding cell adhesion and spreading of hMSCs.

### hMSC Cell Shape is Regulated by Matrix Elasticity

**Figure 2:**
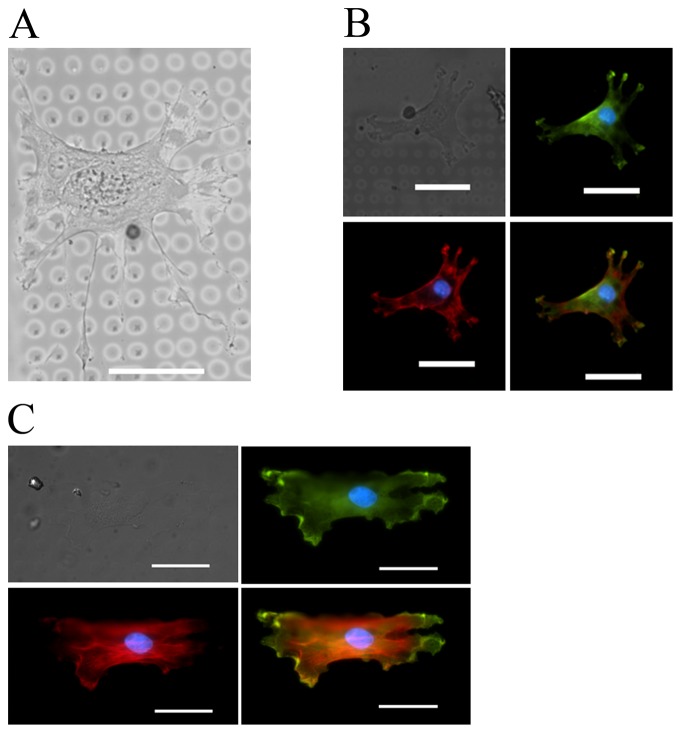
Hydrogel island elasticity regulates cell adhesion and spreading size in MSCs. (A) MSC adhered to pattern with 7 kPa islands, 12 kPa background elasticity, 3 µm spacing, and 100 µg ml^−1^ FN with a cell area of 2,669 µm^2^. Shown as brightfield image (top left), vinculin staining (top right), F-actin staining (bottom left), and merged image (bottom right). Average cell area for data point was 1174.54 µm^2^±113.16 µm^2^. (B) MSC adhered to pattern with 105 kPa islands, 12 kPa background elasticity, 3 µm spacing, and 100 µg ml^−1^ FN with a cell area of 6,134 µm^2^. Shown as brightfield image (top left), vinculin staining (top right), F-actin staining (bottom left), and merged image (bottom right). Nucleus is shown in blue in all images. Average cell area for these conditions was 5847.13 µm^2^±260.56 µm^2^. All scale bars are 50 µm.

To study the behavior of cell spreading on differing physical cues, hMSCs were plated onto micropatterned coverslips. Early passage hMSCs (< passage 6) were plated at a density of 5,000 cells cm^−2^ and given four hours to allow initial cell adhesion. The cells adapted to the patterned islands according to island elasticity, island spacing, ligand density, and background elasticity as shown in Figure 2A. Cells were not allowed to interact with the patterns over long periods to minimize cell modification of the ECM due to secretion and synthesis of components by the cells in particular to the PDMS background. Using the statistical software program Minitab, we ran a linear regression analysis on the cell data and it was observed that hydrogel island stiffness was the key factor in regulating cell adhesion as seen in Equation (1). Cell areas from each condition were compiled into Minitab to run regression analysis and normalized prior to analyzing.

(Equation 1) 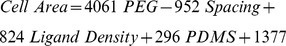



By observing the significance shown by PEG (hydrogel islands) in Equation 1 it is clear that the island adhesion points are the strongest variable controlling cell adhesion. Spacing and ligand density both show reduced efficiency with PDMS (background elasticity) showing insignificant effects.

**Figure 3:**
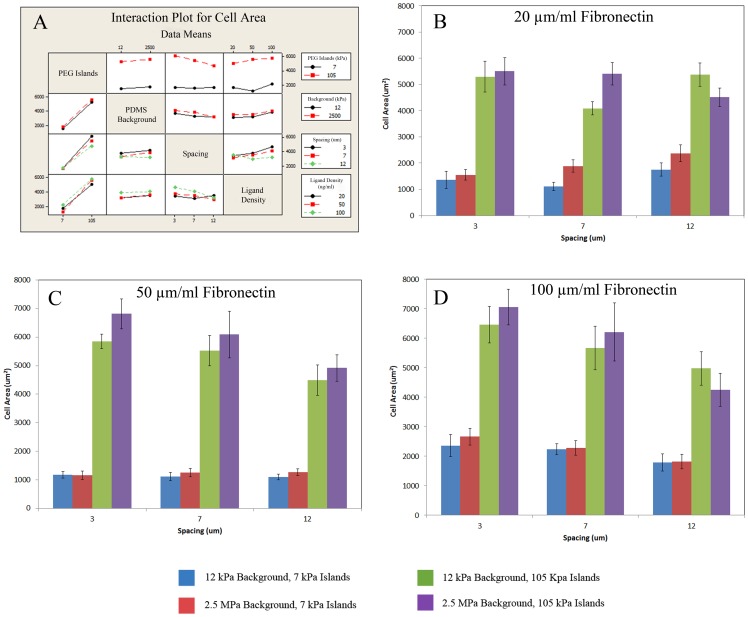
Quantification of average cell area over 36 unique experiments with tunable substrates. (A) The interaction plot illustrates the affects between the non-independent test variables, which include island elasticity, PDMS background ECM elasticity, ligand density, and spacing of islands. Interaction plot uses averages of means to plot interactions. The first column demonstrates the key factor PEG elasticity plays in cell adhesion of MSCs. Spacing and ligand density also are shown to contribute to cell spreading in the interaction plot. PDMS background elasticity was shown to not affect adhesion as shown by PDMS elasticity column showing background elasticity interacting with other variables as nearly parallel lines. Quantification of average cell area for cells with (B) 20 µg/ml, (C) 50 µg/ml, and (D) 100 µg/ml fibronectin concentrations. Error bars are standard error of over 10 cells quantified per condition.

At an island elasticity of 7 kPa, cells preferentially showed a spindle shaped cell orientation similar to myoblasts [29] with smaller cell areas (Figure 2B), while 105 kPa islands were larger, well spread cells similar to osteoblasts [17] (Figure 2C). Figure 3A shows the dependence of cell spreading on island elasticity with stark contrasts in 7 kPa elasticity and 105 kPa elasticity in each condition (P<0.002). Interestingly, when looking at background ECM elasticity for both 7 kPa and 105 kPa islands each case was deemed statistically insignificant to cell area (P>0.05). The interaction plot in Figure 3A illustrates the heavy influence of island elasticity, less significant effects of ligand density and island spacing, and insignificant influence of background matrix.

Island spacing was shown to have smaller effects on cell adhesion. Controls were done with non-patterned hydrogel cover slips and compared to patterned cell areas with equal ligand density and elasticity. The results showed cell areas were significantly altered at 12 µm spacing (P<0.05) when compared to unpatterned controls except for a single condition on 7 kPa PEG. 7 µm spacing also proved significant at the two lower ligand densities (P<0.05) when compared to controls except for a single condition of 105 kPa PEG (Figure 3B-C). In observing 7 and 12 µm spacing, it is evident that without the aid of increasing ligand density for cell adhesion, this is not optimal for cell spreading when compared to its unpatterned counterpart. 3 µm spacing remains significant at lower densities to controls but was deemed insignificant at 100 µg ml^−1^. This result was unsurprising due to the increased adhesion area for cells to attach and continue spreading. At both 20 and 50 µg ml^−1^ FN concentration the 3 µm spacing is significant in 7 kPa PEG on both 12 kPa and 2.5 MPa PDMS backgrounds and 105 kPa on 12 kPa PDMS background (P<0.05) (Figure 3B-C). The 105 kPa PEG on 2.5 MPa PDMS background matrix was not significant in the 20 or 50 µg ml^−1^ conditions (P>0.05). As ligand density was increased it proved to negate spacing effects as evidenced in the 100 µg ml^−1^ FN with 3 and 7 µm cases being deemed insignificant (P>0.05) in each elasticity condition for PEG and PDMS (Figure 3D). Thus, we observed that higher ligand density per island was able to increase cell adhesion area even when distance between islands was increased.

Ligand density was compared at equal conditions for the 100 µg ml^−1^ and 20 µg ml^−1^ FN to observe affects. Differing ligand densities at 3 µm spacing was shown to be statistically relevant in promoting different cell areas except for a single case with 105 kPa PEG. For example, when comparing 7 kPa islands with 100 µg ml^−1^ FN cell area was 2666.04±284.38 µm^2^ to 20 µg ml^−1^ FN and a cell area of 1548.92±203.05 µm2 (P<0.003) (Figure 3D). When observing 7 µm spacing the effects of ligand density diminish, but remain noteworthy at two specific 105 kPa and 7 kPa island test cases (P<0.05). The 7 kPa islands proved the most significant with a cell area of 2234.94±187.0 µm^2^ at 100 µg ml^−1^ compared with 1110.42±159.26 µm^2^ at 20 µg ml^−1^ FN concentration (P<0.0001). As the spacing of the islands increases to 12 µm it was shown to lose statistical relevance. Interestingly, these results show that when only looking at ligand density it has an effect on cell adhesion at smaller spacing and diminishes as spacing is increased. We hypothesize this is due to the amount of adhesive area being greatly reduced at this large spacing, cells were unable to stretch across the same amount of islands rendering the FN concentration insignificant.

Adhesion-mediated signals are shown to be vital in cell-ECM interactions and guiding cell spreading and size. Other reports have used patterned and unpatterned ECMs to guide cell adhesion on differing gel or PDMS surfaces [4], [17], [62]. These studies generally show a consensus for a plateau of cell spreading over approximately 40 kPa. Our results coincide with these other reports and further show the dependence of cell spreading on matrix elasticity when in the presence of other physical factors affecting hMSC spreading. Furthermore, cell generated forces must act in equilibrium, therefore the soft hydrogel islands provide less resistance to a cell's forces and cell contractility decreases. In contrast, stiff islands are able to provide the necessary counterbalancing forces, intracellular tension is increased leading to well spread cells.

### Simulation Predictions

**Figure 4:**
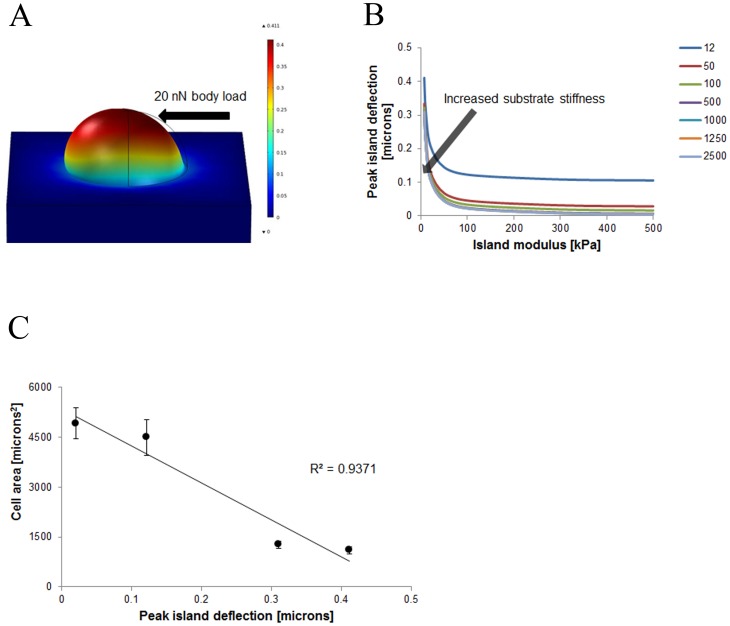
Patterned hydrogel islands were analyzed to engineer substrate elasticity. (A) Conceptual illustration of horizontal cell traction force of 20 nN on hydrogel island and analysis of deflection of individual island. (B) Hydrogel island deflection is plotted as a function of island modulus with differing background elasticities plotted. (C) Cell area of 12 µm spacing cases plotted versus correlating model peak deflections to show correlation between modeling and experimental components.

The mechanical behavior of micropatterned cover slips was characterized with finite-element modeling of the deformation response to cell-derived forces. In all examined cases, PEG islands exhibited significantly greater deflection as compared to the PDMS substrate as seen in Figure 4A. As expected, the greatest deflection occurred when both the island and substrate had the lowest elastic moduli in the examined range. Increasing the PEG island stiffness resulted in a nonlinear decrease in the peak deflection, irrespective of the stiffness of the underlying substrate. Increasing the substrate stiffness had a comparatively diminished effect on the peak island deflection, particularly when the substrate modulus exceeded that of the PEG island shown in Figure 4B. The predicted peak island deflection inversely correlated with the cell area following seeding on micropatterned substrates, suggesting that rigid regions-of-contact between the cell and material facilitate cell spreading seen in Figure 4C.

### Rho Kinase Inhibition Attenuates Differences in Hydrogel Island Mediated Cell Spreading

**Figure 5:**
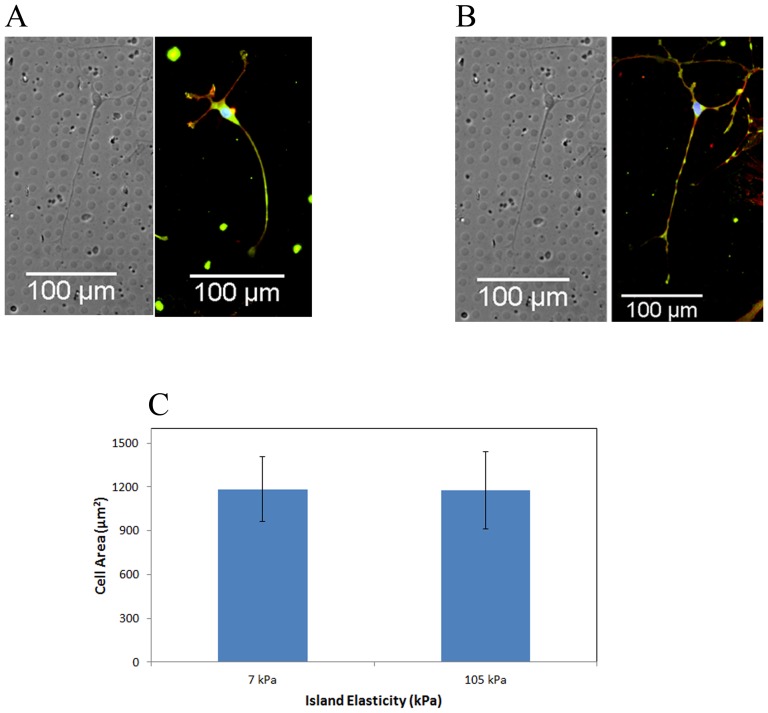
ROCK inhibited cells lose ability to sense matrix conditions. Cells were treated for 7 days, which prevented cells from sensing matrix conditions and spreading as was previously found. (A) ROCK inhibited cell on pattern of 7 kPa PEG, 2.5 MPa background elasticity, 7 µm spacing, and 50 µg ml^−1^ ligand density with brightfield image and vinculin, F-actin, and nucleus staining merged image. Average cell area for this ROCK inhibited trial was 1184.37 µm^2^±223.84 µm^2^. (B) ROCK inhibited cell on pattern of 105 kPa PEG, 2.5 MPa background elasticity, 7 µm spacing, and 50 µg ml^−1^ ligand density with brightfield image and vinculin, F-actin, and nucleus staining merged image. Average cell area for this ROCK inhibited trial was 1175.46 µm^2^±265.79 µm^2^. Error bars are standard error of over 10 cells quantified per condition.

**Figure 6:**
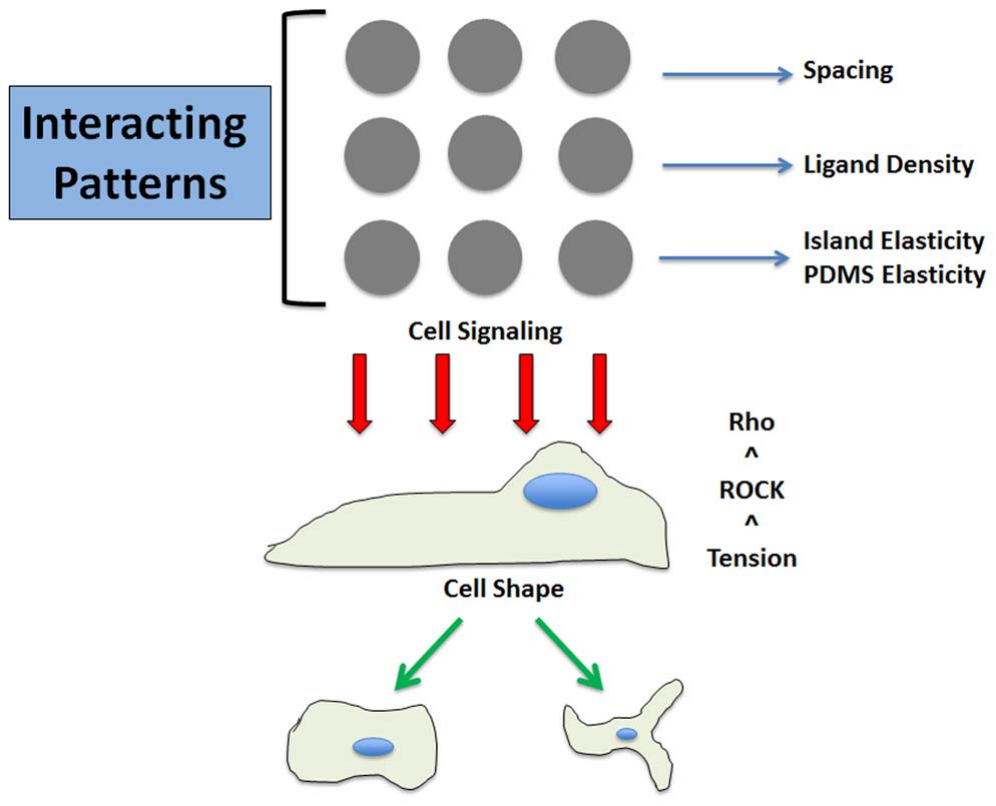
Schematic of mechanical decision made in hMSC commitment. Mechanical cues coordinate to drive hMSC cell shape with RhoA signaling. Interference with cytoskeletal tension disrupts this decision showing the RhoA-ROCK pathway appears critical in adhesion properties of hMSCs.

RhoA has been shown to affect cell size and shape previously as well as play a significant role in cytoskeletal tension in the cell [34], [36]. To address this factor, myosin-generated cytoskeletal tension was inhibited by culturing hMSCs in the presence of Y-27632, an inhibitor of Rho-associated protein kinase (ROCK) that acts as a downstream Rho protein involved in myosin activation. Cells exhibited elongated neuron-like spindles after treatment with Y-27632 on both 7 kPa islands and 105 kPa islands with no change in regards to patterned island elasticity as shown in Figure 5A and Figure 5B. 7 kPa island elasticity cell area averages were 1184.37±223.84 µm^2^ while 105 kPa island cell areas were 1175.46 µm^2^±265.79 µm^2^. Integrins and focal adhesions are the binding point of cells to the ECM and our results confirm that this tension sensing occurs through this RhoA signaling pathway [79], [80], [81]. Focal adhesions transmit force to the actin cytoskeleton causing it to remodel according to physical cues and it is able to alter cell size and shape as seen in the schematic in Figure 6. Thus, ROCK inhibited cells were confirmed to lose the ability to sense matrix elasticity when myosin contractions were suppressed demonstrating the background elasticity is unimportant and confirms that RhoA plays a prominent role in sensing matrix stiffness.

## Conclusions

In summary, our experimental and modeling findings showed matrix elasticity to be the key regulator of hMSC adhesion on surfaces with independently tunable physical and chemical properties. Cell spreading area was predominantly controlled by matrix elasticity with soft matrices showing smaller cells and stiff matrices showing large cells. Our modeling component was able to display a high degree of correlation between cell spreading and island deflection showing how softer hydrogel islands lead to reduced cell spreading and thus confirming our experimental data. In controlling the ECM characteristics and parsing cooperative signaling pathways, we hope to gain a better understanding of the interactions between cell-ECM interactions and further cell behavior such as lineage commitment. By combining a modeling and experimental component we can gain further understanding and confidently utilize finite element modeling as a predictive tool in analyzing cell function and behavior. This will potentially have great implications in the field of stem cell engineering and regenerative medicine such as optimizing the characteristics of scaffolds and inducing homogenous populations of lineage committed cells.
